# Transcranial Magnetic Stimulation Reveals Executive Control Dissociation in the Rostral Prefrontal Cortex

**DOI:** 10.3389/fnhum.2017.00464

**Published:** 2017-09-22

**Authors:** Weijiang He, Chenggui Fan, Ling Li

**Affiliations:** Key Laboratory for NeuroInformation of Ministry of Education, High-Field Magnetic Resonance Brain Imaging Key Laboratory of Sichuan Province, Center for Information in Medicine, School of Life Science and Technology, University of Electronic Science and Technology of China Chengdu, China

**Keywords:** rostral prefrontal cortex, prospective memory, working memory, transcranial magnetic stimulation, executive control

## Abstract

Although previous studies have shown that the rostral prefrontal cortex (rPFC) plays a crucial role in executive tasks, the various functions of the rPFC in the humans are still understudied. Here we used transcranial magnetic stimulation (TMS) with continuous theta burst stimulation (cTBS) to interfere with the executive control functions of the right rostrolateral PFC (RLPFC) or the right rostromedial PFC (RMPFC). Subjects performed a task-switching paradigm, which included spatial detection (SD), prospective memory (PM) and working memory (WM) tasks, after cTBS. The performance of 18 healthy volunteers was evaluated on different days after cTBS over the right RLPFC, the right RMPFC, and the vertex (serving as a control site). The application of cTBS over the RLPFC significantly increased the switching costs (SCs) of the error rates (ERs) when switching to the PM task, while RMPFC-cTBS decreased SCs of ERs when switching to the WM task, compared with the control vertex site. These findings provide evidence for a differential role of the RLPFC and the RMPFC in executive functions, with a specific involvement of the RLPFC and the RMPFC in PM, and WM, respectively.

## Introduction

The rostral prefrontal cortex (rPFC, Brodmann Area 10), which is the largest and most anterior region within the prefrontal cortex (PFC), is reported to play a crucial role in several cognitive processes and executive functions such as prospective and working memory (WM; Ramnani and Owen, 2004; Gilbert et al., 2006; Burgess and Wu, 2013). Over the past decades, many clinical and experimental tests have been used to evaluate executive functions. Recent evidence from a lesion study demonstrated that deficits in a number of executive tasks were associated with damage of the right rPFC (Roca et al., 2010). These deficits have been suggested to be linked by several common cognitive processes such as multitasking and the ability to switch between cognitive contexts (Burgess et al., 2000; Koechlin and Summerfield, 2007; Badre and D’Esposito, 2009). However, the evidence regarding the specific functions of the rPFC in humans remains controversial.

Prospective memory (PM), which is one element needed for successful multitasking, is the ability to remember to carry out intentions at the appropriate time in the future; for example, remembering to take a medication at a particular moment or to refuel the car. This function involves the formation, maintenance, updating, retrieval and execution of intention (Basso et al., 2010; Cona et al., 2015). In a typical PM experiment, participants are required to complete the intended PM actions when a specific target cue appears or upon the arrival of a particular time point, while engaging in the ongoing task (Chen et al., 2015). Functional neuroimaging studies have shown widespread activation of the rPFC during the maintenance of intentions (Burgess et al., 2001, 2003; Semendeferi et al., 2001; Gilbert et al., 2009; Reynolds et al., 2009). Recent evidence from lesion studies have suggested that rPFC damage leads to PM deficits (Uretzky and Gilboa, 2010; Umeda et al., 2011; Volle et al., 2011; Szczepanski and Knight, 2014). In addition, a transcranial magnetic stimulation (TMS) study has provided evidence for the critical role of rPFC in PM (Costa et al., 2011).

The role of the rPFC in WM has been continuously studied over the past decades. WM is a limited capacity cognitive system for the temporary storage and manipulation of remembered information so that the central executive system is responsible for allocating resources to manage the maintenance of relevant information while suppressing task-irrelevant information (Baddeley, 2003, 2010). Several neuroimaging and neuropsychological studies have reported that the rPFC is a critical region during the performance of WM tasks (Christoff and Gabrieli, 2000; Braver and Bongiolatti, 2002; Badre and Wagner, 2004; Volle et al., 2005). This has been further supported by a meta-analyses study, which showed that a variety of n-back tasks provoked activation in the bilateral rPFC (Owen et al., 2005). More recently, a functional magnetic resonance imaging (fMRI) study using endogenous PM paradigm involving incremental updating of WM has reported activation of the left rPFC during the performance of WM (Halahalli et al., 2014).

Furthermore, based on neuroimaging findings, there is some evidence of a functional specialization within the rPFC across the lateral-medial axes (Gilbert et al., 2006; Burgess et al., 2007). The lateral aspects of rostral PFC (RLPFC) tend to exhibit robust activation during the maintenance of an intention over a delayed period (Burgess et al., 2001, 2003; Simons et al., 2006; Gilbert et al., 2009; Reynolds et al., 2009; Okuda et al., 2011). On the flip side, the medial aspects of the rostral PFC (RMPFC) demonstrate increased activity during the performance of a WM task relative to PM conditions (Okuda et al., 2007; Hashimoto et al., 2011). However, the functional specialization within the rPFC remains poorly understood. In the present article, we therefore undertake TMS to investigate functional specialization within the rPFC.

The objective of the present study was to investigate the executive control functions associated with the medial and lateral rostral prefrontal cortex during the performance of a PM and a WM task. We used continuous theta burst stimulation (cTBS), which is a special type of repetitive TMS (rTMS), to temporarily disrupt the function of the right RLPFC and the right RMPFC, compared to a control region. A task-switching paradigm was used, which included spatial detection (SD), PM and WM tasks. The three different tasks were displayed in a pseudo-random order. Previous findings show that when participants switch between the tasks, reaction times (RTs) become longer and error rates (ERs) increase, a phenomenon known as “switch costs” (Li et al., 2012). Switch costs (SCs) represent the difference between the performance of switched trials and repeated trials (Bahlmann et al., 2015). Thus, we hypothesized that, compared to the control region, cTBS over the RLPFC would increase switch costs during the PM task, while cTBS over the RMPFC will disturb the performance of the WM task.

## Materials and Methods

### Participants

Eighteen healthy volunteers were enrolled (9 females, mean age 22.94 ± 2.66 years) from the University of Electronic Science and Technology of China. All participants were right-handed, had normal or corrected-to-normal visual acuity and normal color vision. The standard TMS exclusion criteria were used, including pregnancy, a metallic implant, a cardiac or neurological health condition, and the intake of a specific medication. No participants reported a neurological history (including epilepsy), psychiatric disorders, or drug abuse. All participants gave their informed written consent before participating and were paid for their attendance. The cTBS session was performed according to the published safety guidelines (Wassermann et al., 1996; Rossi et al., 2009). This study was approved by the local committee for the Protection of Human Subjects for the University of Electronic Science and Technology of China. The methods were carried out in accordance with the approved guidelines and all experiments conformed to the declaration of Helsinki.

### Stimuli and Task

**Figure 1:**
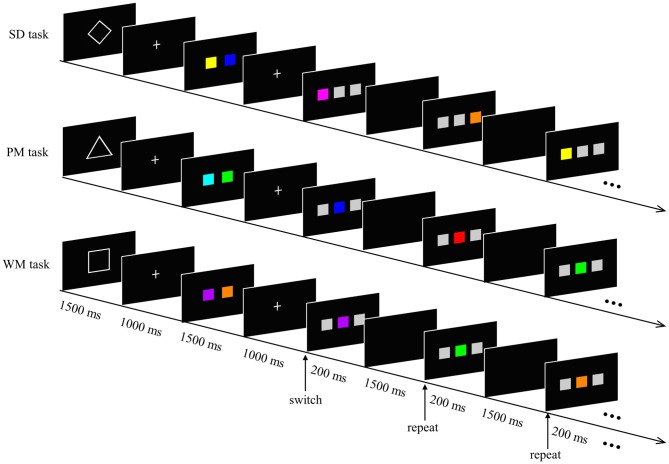
Experimental paradigm. The experimental task was composed of a spatial detection (SD) task, prospective memory (PM) task and working memory (WM) task. A 1500 ms geometric shape (diamond, triangle and square for SD, PM and WM, respectively) signaled the task type. Two 1000 ms fixation crosses were separated by a 1500 ms display of two colored squares. Subsequently, five sequential response trials of the same type were presented for 200 ms (each separated by a blank black screen for 1500 ms). Subjects responded to each trial according to the current task directions. In the SD task, participants had to detect the spatial position of the colored square, regardless of the color. In the PM task, a prospective intention (red square) was encoded before the experiment started. Participants responded when they encountered the red colored square. In the WM task, participants had to decide whether the current colored square matched one of the two memorized colored-squares or not (for details, see text).

We used a modified version of the task-switching paradigm (Henseler et al., 2011). The experimental task was composed of three different short tasks: a SD task, a PM task and a WM task (see Figure 1). At the beginning of each task, a cue (geometric shape) that informed subjects about which task they had to perform was presented for 1500 ms. This was followed by two displays: one showing a white crosshair on the black center of the screen for 1000 ms, and another display showing two colored squares for 1500 ms. Subsequently, five sequential response trials of the same type were presented for 200 ms (each separated by a blank black screen for 1500 ms). According to the current task directions, subjects had to respond to each trial by pressing the “F” or “J” key on a PC keyboard with their left or right index finger, respectively. In each of these trials, three squares were displayed in a horizontal row, where only one of the squares was colored and the others were gray. The experiment consisted of a total of nine different colors (including gray) with equal luminance and saturability. The red color only appeared during the PM task.

In the SD task, the colored square appeared either on the left or the right position on the screen. In other words, the two gray squares were always adjacent and were located either to the left or the right of the colored square. Participants had to detect the spatial position of the colored square and were instructed to press the “F” key with their left index finger as quickly as possible when the colored square appeared on the left position, and to press the “J” key with their right index finger when the colored square occurred on the right position.

During the PM task, the colored square was presented between the two gray squares. Participants were informed of the target (red square) and asked to respond with their left index finger when the target appeared, and with their right index finger when the square was shown in any other color.

During the WM task, the colored square was still displayed in the center of the two gray squares. However, here the participants had to remember the two colored-squares, which were presented at the beginning of the short task. The participants had to decide whether the current colored square matched one of the two memorized colored-squares or not. Participants were instructed to make a response with their left index finger if the current stimulus matched one of the memorial items, and to to respond by pressing with their right index finger if it did not match these items.

E-prime (Psychology Software Tools, Pittsburgh, PA, USA) was used for stimulus presentation and recording of the behavioral results.

### Experimental Procedures

Each session included four runs (each lasted 5 min and 24 s) with a short break between runs. Each of the runs consisted of three kinds of tasks of eight blocks. The order of the tasks was pseudo-randomized. The first trial was considered a “repeat trial” if the current task was identical to the preceding one, otherwise it was considered a “switch trial”. Each participant had 480 trials in total, 15.2% of which were switch trials. The participants completed the three sessions of the task on three separate days, so that on each day a different single site was stimulated using cTBS. The order of the stimulation sessions was counterbalanced across the participants. In order to make familiarize the participants with the behavioral task, each performed a brief training session before the beginning of each experimental session. Each subject received TMS to one of three different sites before performing the same experimental task.

### TMS Protocol and Stimulation Sites

cTBS, a special type of rTMS, was performed using a Magstim super rapid^2^ magnetic stimulator (the Magstim Company Limited, Whiteland, UK), equipped with an air-cooled figure-of-eight coil having an outer winding diameter of 70 mm. We used the following cTBS parameters: 50 Hz trains of three biphasic pulses that were repeated every 200 ms (5 Hz), and a continuous stimulation for 30 s (450 pulses in total) at 80% of the active motor threshold (AMT; Huang et al., 2005). Before the cTBS session, we determined the AMT in each participant using a previously described method (Rossini et al., 1994). The mean stimulation intensity of the cTBS was 31 ± 3.4% (mean ± SD) of the maximum machine output. Since participants may experience a degree of discomfort or even pain when cTBS is applied over the rostral prefrontal cortex, we delivered a test pulse prior to the experimental session and asked the participants whether they felt the pulse aversive. All the participants tolerated the cTBS well and did not ask to stop the experiment nor did they pull their head away from the coil during the stimulation (Ryals et al., 2015).

**Figure 2:**
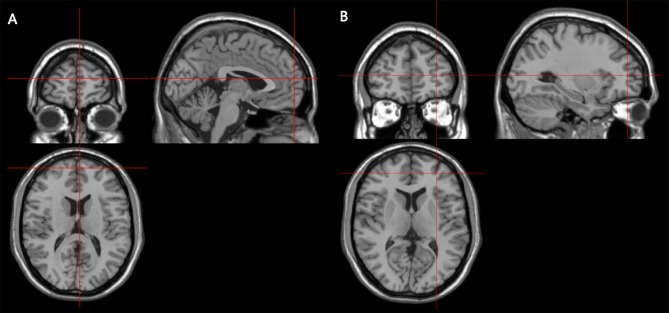
Coronal, axial and sagittal views of the stimulated sites, as depicted on a standard template from MRIcro. **(A)** The MNI coordinates of the right rostromedial PFC (RMPFC) [3 60 15], corresponding to right medium frontal gyrus in the rostral prefrontal cortex. **(B)** The MNI coordinates of right rostrolateral PFC (RLPFC) [30 48 9], corresponding to right superior frontal gyrus in the rostral prefrontal cortex.

Stimulation locations were targeted via the BrainSight stereotaxic neuronavigator (Rogue Research, Montreal, QC, Canada), equipped with a Polaris Vicra position sensor system. Landmarks on all participants’ head were co-registered to a standard MRI template using MNI (Montreal Neurological Institute) coordinates. The stimulation sites were determined on the basis of the coordinates of the activation peaks from a previous fMRI study (Henseler et al., 2011), which used behavioral tasks similar to those used in our study. The locations of the two target stimulation sites were centered on the following MNI (Montreal Neurological Institute) coordinates: the right rostromedial PFC (RMPFC) was [3 60 15] (SEM for 0.47, 0.70, 0.47 mm), the right rostorlateral PFC (RLPFC) was [30 48 9] (SEM for 0.70, 0.94, 0.47 mm; see Figure 2). The vertex, which served as the control site, was localized as the midpoint between the inion and the nasion and equidistant from the left and right ear. The TMS coil was placed on the corresponding locations over the participants’ scalp. Brainsight was used to track the position of the TMS coil throughout the stimulation period, ensuring that it remained on the target location.

### Data Analyses

The ERs and mean RTs of the correct responses were analyzed separately using SPSS software (version 22, SPSS Inc., Chicago, IL, USA). Trials with RTs either faster than 200 ms or slower than 1000 ms were deemed false responses. First, each of the repeated measure analysis of variance (ANOVA) of the ER and the RT was performed using three independent factors: TMS sites (three levels: RLPFC, RMPFC and vertex), task type (three levels: SD, PM and WM) and task modality (two levels: repeat and switch). Subsequently, repeated-measures ANOVAs for ERs and RTs, using within-subject factors of TMS sites (three levels: RLPFC, RMPFC and Vertex) and task type (three levels: SD, PM and WM), were performed separately for switch and repeat trials. Switch costs were defined as the ERs or RTs difference between switch trials and repeat trials (Bahlmann et al., 2015). The comparisons of the ERs and RTs switch costs were performed using ANOVAs with the following within-subject factors: TMS sites (three levels: RLPFC, RMPFC and Vertex) and task type (three levels: SD, PM and WM). *Post hoc t*-tests were corrected using the Bonferroni correction for multiple comparisons and corrected *p* values of less than 0.05 were deemed statistically significant.

## Results

Table 1 displays the mean values of the ERs and RTs for each TMS site.

**Table 1:** Mean values (and SEM) of Error Rates (ERs) and Reaction Times (RTs).

TMS site	Task type	Task modality	Error rates (%)		RTs (ms)	
			Mean	SEM	Mean	SEM
RLPFC	SD	repeat	2.673	0.954	351.403	7.714
		switch	1.235	0.519	395.234	11.748
	PM	repeat	4.136	0.82	383.861	11.251
		switch	10.386	1.993	454.085	20.57
	WM	repeat	11.679	1.557	446.347	12.816
		switch	10.87	1.835	486.05	20.642
Vertex	SD	repeat	1.646	0.372	360.702	9.078
		switch	1.111	0.542	397.98	12.091
	PM	repeat	3.704	0.911	394.647	11.292
		switch	4.798	1.298	459.354	17.983
	WM	repeat	9.784	1.687	460.936	13.601
		switch	10.684	1.98	485.656	16.482
RMPFC	SD	repeat	2.068	0.542	356.067	8.44
		switch	0.725	0.527	403.823	13.001
	PM	repeat	3.909	0.900	389.632	11.135
		switch	6.667	1.771	454.295	15.498
	WM	repeat	12.881	2.415	457.505	13.928
		switch	8.222	1.726	490.595	19.465

*RLPFC, rostrolateral prefrontal cortex; RMPFC, rostromedial prefrontal cortex; SD, spatial detection; PM, prospective memory; WM, working memory*.

### Error Rates

The 3 (TMS sites: RLPFC, RMPFC and Vertex) by 3 (task type: SD, PM and WM) by 2 (task modality: repeat and switch) repeated-measures ANOVA comparing ERs showed significant task modality interactions (TMS sites × task type × task modality: *F*_(4,68)_ = 3.534, *p* = 0.011, TMS sites × task modality: *F*_(2,34)_ = 3.574, *p* = 0.039, task type × task modality: *F*_(2,34)_ = 12.987, *p* < 0.001). In addition, there was a significant main effect for the TMS site (*F*_(2,34)_ = 4.995, *p* = 0.0125) and task type (*F*_(2,34)_ = 34.919, *p* < 0.001).

**Figure 3:**
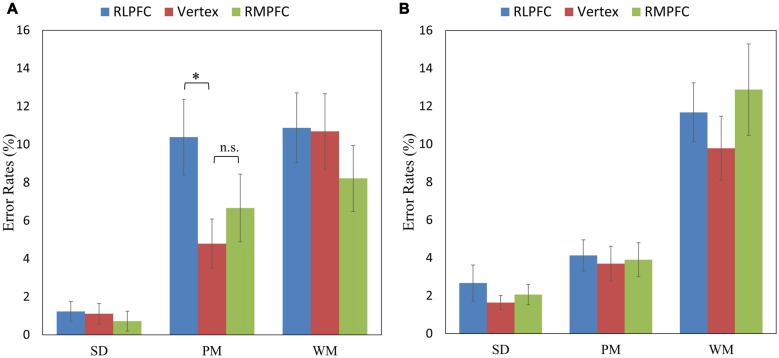
The mean error rates (ERs) of the switch trials (in the left panel **A**) and repeat trials (in the right panel **B**) across the three tasks (SD, PM and WM), and three stimulation sites: RLPFC (blue), vertex (red), RMPFC (green). *p < 0.05, n.s = non-significant. Error lines represent standard error of mean.

Since there was a 3-way interaction, further analyses of the ERs were conducted for switch trials and repeat trials separately. The 3 (TMS sites: RLPFC, RMPFC and Vertex) by 3 (task type: SD, PM and WM) repeated-measures ANOVA comparing ERs during switch trials showed a significant TMS sites by task type interaction (*F*_(4,68)_ = 2.802, *p* = 0.032), a main effect for the TMS site (*F*_(2,34)_ = 5.251, *p* = 0.01) and a main effect for the task type (*F*_(2,34)_ = 23.480, *p* < 0.001). Multiple comparisons showed that ERs were significantly higher after RLPFC-TMS compared with the control site, only switching to the PM task (*t*_(17)_ = 3.341, corrected *p* = 0.039; see Figure 3A).

The 3 (TMS sites: RLPFC, RMPFC and Vertex) by 3 (task type: SD, PM and WM) repeated-measures ANOVA comparing ERs during repeat trials revealed a significant main effect of task type (*F*_(2,34)_ = 41.702, *p* < 0.001). However, the TMS site (*F*_(2,34)_ = 2.777, *p* = 0.076) and the interaction between TMS site and task type (*F*_(4,68)_ = 1.824, *p* = 0.134) were not significant (see Figure 3B).

### Switch Costs of Error Rates

**Figure 4:**
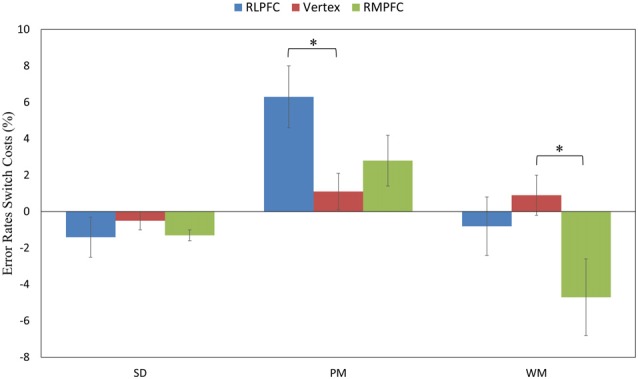
The switch costs of ERs across the three tasks (SD, PM and WM), and three stimulation sites: RLPFC (blue), vertex (red), RMPFC (green). *p < 0.05, error lines represent standard error of mean.

To further assess the difference in performance between the switched and repeated trials, we calculated the ERs switch costs across the TMS sites and task type. The 3 (TMS sites: RLPFC, RMPFC and Vertex) by 3 (task type: SD, PM and WM) repeated-measures ANOVA of the ER switch costs showed a significant TMS site by task type interaction (*F*_(4,68)_ = 3.534, *p* = 0.011), a main effect of TMS site (*F*_(2,34)_ = 3.574, *p* = 0.039) and a main effect of task type (*F*_(2,34)_ = 12.987, *p* < 0.001). *Post hoc* comparisons revealed that ERs switch costs were significantly greater when stimulating the RLPFC site in comparison to the vertex site switching to the PM task (*t*_(17)_ = 2.68, corrected *p* = 0.0338), and the ERs switch costs were significantly smaller when stimulating the RMPFC region in comparison with the vertex switching to the WM task (*t*_(17)_ = 2.89, corrected *p* = 0.01995; see Figure 4). No other comparisons reached significance.

### Reaction Times

A similar analysis was performed for the comparison of the reaction time. The 3 (TMS sites: RLPFC, RMPFC and Vertex) by 3 (task type: SD, PM and WM) by 2 (task modality: repeat and switch) repeated-measures ANOVA revealed a significant interaction between task type and task modality (*F*_(2,34)_ = 10.706, *p* < 0.001), a significant main effect of task type (*F*_(2,34)_ = 119.703, *p* < 0.001) and of task modality (*F*_(1,17)_ = 53.023, *p* < 0.001).

### Switch Costs of Reaction Times

The 3 (TMS sites: RLPFC, RMPFC and Vertex) by 3 (task type: SD, PM and WM) repeated-measures ANOVA comparing RTs switch costs showed a significant main effect of task type (*F*_(2,34)_ = 10.706, *p* < 0.001). However, the main effect for the TMS site (*F*_(2,34)_ = 1.085, *p* = 0.349) and the interaction between TMS site and task type (*F*_(4,68)_ = 0.558, *p* = 0.694) were non-significant.

## Discussion

The objective of the current study was to investigate the relationship between the medial and lateral rostral prefrontal cortex during a PM task and an ongoing WM task by means of cTBS. Our findings show that cTBS over the right rostral prefrontal cortex affected both PM and WM performance, relative to the stimulation of a control region. Specfically, cTBS over the rostorlateral PFC significantly increased subjects’ switching costs when switching to the PM task. Conversely, switching costs significantly decreased following cTBS over the rostromedial PFC when switching to the WM task.

To make our participants be familiar with the behavioral task, each participant first performed a brief training session before the beginning of each experimental days. Then, participants performed a session without cTBS stimulation, the no-cTBS session, followed by the cTBS stimulation (right RLPFC, right RMPFC or the vertex). Finally, the participants completed a session until the end of the experimental task. We performed a 3 (Time: day1, day2, day3) by 3 (task type: SD, PM and WM) by 2 (task modality: repeat and switch) repeated-measures ANOVA on the ERs of 3 no-cTBS sessions. We found no main effect of the time (*F*_(2,34)_ = 0.268, *p* = 0.758). Therefore, the average value of the three no-cTBS sessions performed on each stimulation day was used as the baseline. The 2 (TMS sites: Baseline and Vertex) by 3 (task type: SD, PM and WM) by 2 (task modality: repeat and switch) repeated-measures ANOVA comparing ERs revealed a main effect for task type (*F*_(2,34)_ = 31.828, *p* < 0.001), but other main effects were not found (all *p* > 0.05, specifically for the TMS sites: *F*_(1,17)_ = 0.66, *p* = 0.428). In order to exclude unspecific TMS effects, we used the stimulation of vertex as the control site.

The first main finding is that the ERs were significantly greater during the performance of the PM task following inhibitory cTBS over the right rostral prefrontal cortex, compared with cTBS over a control region. This finding is consistent with those of previous TMS studies and a body of functional neuroimaging investigations that demonstrated the involvement of the rostral prefrontal cortex (BA 10) in the mediation of PM processes (Burgess et al., 2008, 2011; Costa et al., 2011, 2013; Halahalli et al., 2014). Moreover, consistent with previous evidence, the rostral prefrontal cortex has been implicated in WM and other PM tasks (Ramnani and Owen, 2004; Gilbert et al., 2006; Burgess and Wu, 2013).

The second main finding of our study showed that inhibitory cTBS over the rostorlateral PFC significantly increased subjects’ switching costs when switching to the PM task but not to the WM task. The rostorlateral PFC has been shown to be associated with PM processes and tends to exhibit robust activation during the maintenance of delayed intentions (Burgess et al., 2001, 2003; Simons et al., 2006; Gilbert et al., 2009; Reynolds et al., 2009; Okuda et al., 2011). Our finding is in line with a previous study (Reynolds et al., 2009) showing that the rostorlateral PFC is activated during event-based PM tasks but is not activated when there are WM demands (Burgess and Wu, 2013).

The third main finding of our study showed that inhibitory cTBS over the rostromedial PFC significantly decreased subjects’ switching costs when switching to the WM task but not to the PM task. At first glance, this may seem to be in conflict with other studies demonstrating greater rostromedial PFC activity during an ongoing WM task compared to PM conditions (Okuda et al., 2007; Hashimoto et al., 2011). The RMPFC is part of the default mode network (DMN) and has been known to show task induced deactivation during the performance of demanding cognitive tasks compared to a resting baseline (Sridharan et al., 2008; Buckner et al., 2009). Recently, a study shows the RMPFC exhibited activation during a task preparation period but deactivation during a task execution period (Koshino et al., 2011). The most probable explanation for this discrepancy between our finding and previous studies is that the RMPFC was reversely activated after cTBS stimulation.

In conclusion, here we investigated the differential role of the lateral and medial rostral prefrontal cortex in PM and WM, respectively. Our findings support the notion that the rostral PFC plays a key role in both PM and WM. Importantly, our findings demonstrate a functional specialization within the rostral PFC along a lateral-medial dimension, with the rostorlateral PFC being preferentially involved in PM and the rostromedial PFC in ongoing WM.

## Author Contributions

WH performed research, analyzed data and wrote the main manuscript text. CF revised the manuscript. LL designed research. All authors reviewed the manuscript.

## Conflict of Interest Statement

The authors declare that the research was conducted in the absence of any commercial or financial relationships that could be construed as a potential conflict of interest.
